# The complete mitochondrial genomes of *Puccinia striiformis* f. sp. *tritici* and *Puccinia recondita* f. sp. *tritici*

**DOI:** 10.1080/23802359.2019.1674744

**Published:** 2019-12-09

**Authors:** Chuang Li, Xianhua Lu, Yibo Zhang, Na Liu, Chunqi Li, Wenming Zheng

**Affiliations:** Collaborative Innovation Center of Henan Grain Crops / State Key Laboratory of Wheat and Maize Crop Science, College of Life Sciences, Henan Agricultural University, Zhengzhou, China

**Keywords:** Rust fungi, mitochondrial genome, *Puccinia striiformis*, *Puccinia tritici*

## Abstract

The complete mitogenomes of *Puccinia striiformis* f. sp. *tritici* (*Pst*) CY32 and *P. recondita* f. sp. *tritici* (*Pt*) HnZU18-3, two destructive rust fungi in wheat, are sequenced and determined. Their circular mitogenomes are 102,521 bp and 77,894 bp in length and contain 24 and 23 tRNA genes for *Pst* and *Pt*, respectively, and both consist of 14 protein-coding genes located on the same strand. Phylogenetic analysis revealed the phylogenetic relationships of *Pst* and *Pt* based on mitogenomes conform to the previous identification in genomic level.

*Puccinia striiformis* f. sp. *tritici* (*Pst*) and *Puccinia recondita* f. sp. *tritici* (*Pt*), members of most prevalent plant pathogenic fungi, cause wheat stripe rust and leaf rust across the world (Huerta-Espino et al. 2011; Chen et al. 2014). They belong to the order Pucciniales and the family Pucciniaceae. Many versions of genome assemblies of *Pst* and *Pt* have been available on the NCBI website. The complete sequences of their compact mitogenomes would provide more insights into further genomic study of rust fungi.

In this study, we collected urediniospores of *Puccinia striiformis* f. sp. *tritici* CY32, obtained from Kang Lab in Northwest A&F University of China, and *Puccinia recondita* f. sp. *tritici* HnZU18-3, sampled from Henan province of China. Both of the isolates were identified for their virulence and purified on wheat cultivar MX169 in greenhouse. The collected urediniospores were stored in a refrigerator at 4 °C and the total DNA was extracted using a modified CTAB method. *Pst* DNA was sequenced using PacBio RsII and Illumina Hiseq 2000. *Pt* DNA was sequenced using PacBio Sequel I and Illumina NovaSeq 6000. Mitochondrial sequences were identified using fungal mitochondrial sequences. Long reads were used for *de novo* assembly using Miniasm (Li 2016), followed by error correction using short reads. We identified their circularization using mapping long reads to the assemblies. PCR verification was performed to evaluate the correctness. The final mitogenomes of *Pst* and *Pt* are 102,521 bp and 77,894 bp in length, respectively, and have been deposited under the GenBank accession numbers MH891489 and MN004749, respectively.

The mitochondrial base composition for *Pst* and *Pt* is A 35.46%, T 33.14%, C 11.07%, G 20.33% and A 34.60%, T 27.97%, C 13.42%, G 24.01%, respectively. We predicted 14 protein-coding genes (atp6, atp8, atp9, cob, cox1, cox2, cox3, nad1, nad2, nad3, nad4, nad4L, nad5, and nad6) located on the same strand for *Pst* and *Pt*. 24 and 23 tRNA genes on the same strand were identified using tRNA scan-SE (Lowe and Chan 2016) for *Pst* and *Pt*, respectively.

The two mitogenomes are highly syntenic using mitogenome alignment. In order to infer the phylogenetic relationship of *Pst* and *Pt* based on mitogenomes, we constructed the maximum-likelihood tree based on concatenated amino acids using Mega-X (Kumar et al. 2018) with 1000 bootstrap replicates under WAG + G + F model. The phylogenetic tree (Figure 1) shows that the topology is under high support and the phylogenetic relationships of *Pst* and *Pt* are consistent with theirs in genomic level (Zheng et al. 2013; Cuomo et al. 2017).

**Figure 1.**
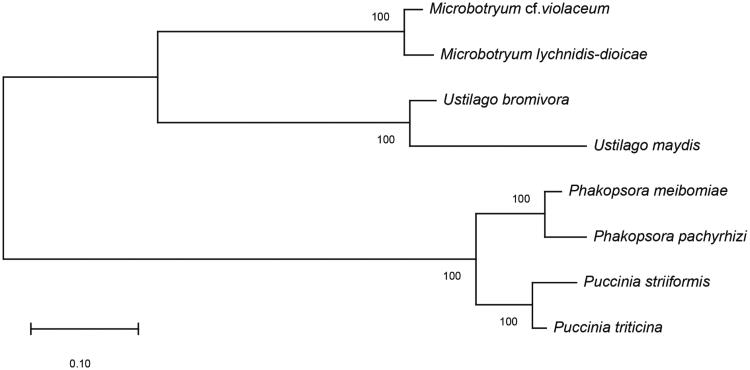
Phylogenetic relationship of eight species among Pucciniomycotina and Ustilaginomycotina based on concatenated amino acids (atp6-atp8-atp9-cob-cox1-cox2-cox3-nad1-nad2-nad3-nad4- nad4L-nad5-nad6). The selected species include: *Puccinia striiformis* (MH891489), *Puccinia tritici* (MN004749), *Phakopsora pachyrhizi* (NC_014344), *Phakopsora meibomiae* (NC_014352), *Microbotryum lychnidis*-*dioicae* (NC_020353), *Microbotryum* cf. *violaceum* (NC_020354), *Ustilago maydis* (NC_008368), *Ustilago bromivora* (LT558140).
